# Using Expert Elicitation and Systems Mapping to Support Social Innovation in the (Blue) Food System: The Case of the UK's Plymouth Fish Finger

**DOI:** 10.1111/jhn.70234

**Published:** 2026-03-23

**Authors:** Pettinger Clare, Parsons Kelly, Irazu Yanaina Chavez‐Ugalde, Hunt Louise, Wagstaff Carol

**Affiliations:** ^1^ School of Health Professions, Faculty of Health University of Plymouth Plymouth UK; ^2^ MRC Epidemiology Unit University of Cambridge Cambridge UK; ^3^ Centre for Food Policy City St George's, University of London London UK; ^4^ Department of Food and Nutritional Sciences University of Reading Reading UK

**Keywords:** blue food system transformation, causal loop diagram, collaborative processes, expert elicitation, policy, social innovation, systems mapping, systems thinking

## Abstract

**Background:**

The imperative for food system transformation is well known, yet to date there has been minimal emphasis on the blue food system [foods sourced from marine and freshwater environments]. Generally, a food systems approach should shift away from linear and move towards more systems thinking to embrace complexity. This paper focuses on a local social innovation project (Plymouth Fish Finger (PFF)) which has pioneered localising the blue food system. This study aimed to elicit how the (policy and practice) system around the PFF can be appraised to optimise social innovation practices for (blue) food system transformation.

**Methods:**

Expert elicitation combined with group model building (GMB) to co‐create and validate a ‘Causal Loop Diagram’ (CLD) to visually understand the policy and practice implication and needs of the PFF initiative. Purposive sampling to recruit a range (*n* = 14 total) of experts representing the different parts of the system. Two ‘mapping’ workshops (one face‐to‐face, one online) facilitated elicitation of expert input into the process to enable establishment of a final synthesised systems map for critique and validation.

**Findings:**

Hand‐created maps evolved into a validated CLD, containing 49 elements connected by 130 causal links and 5 feedback loops. These loops revealed how demand generation, supply chain capacity, economic viability, trust and product consistency, and infrastructural constraints, reinforce or balance system performance. Six themes emerged: (i) demand generation, (ii) supply chain constraints, (iii) economic viability, (iv) social innovation and trust, (v) nutritional guidance and (vi) unintended consequences. The CLD also enabled interventions to be pinpointed within a system to inform policy/practice actions for change.

**Conclusions:**

We illustrate how systems thinking and expert elicitation approaches have successfully encouraged dynamic dialogue to support the identification of future policy and practice interventions. This demonstrates how social innovation projects can be championed and their powerful potential for catalysing (blue) food system transformation better realised.

## Summary

- Food system transformation requires a shift away from linear towards more ‘systems thinking’ to embrace complexity.
- This paper uses a local social innovation project (The Plymouth Fish Finger (PFF)) as an exemplar to elicit how policy and practice can be appraised to catalyse blue food system change.
- Expert elicitation and group model building were used to enable interactive mapping and co‐creation of a causal loop diagram which was validated by a range of blue food experts.
- These approaches successfully encouraged dynamic dialogue to support the identification of future policy and practice interventions.
- Recommendations are made for a range of policy and practice actions and future research potential.

## 1 Introduction

The imperative for food system transformation is well known, and concerted action is essential to impact inter‐related food system issues [1]. Food systems discourses to date have focussed mainly on livestock and land‐based agriculture [2], with little emphasis placed on transforming the system around ‘blue foods’ defined as ‘fish, invertebrates, algae and aquatic plants captured or cultured in freshwater and marine ecosystems’ [3]. Recent calls for a global ‘Blue Transformation’ from the United Nations [4] and the Eat Lancet Commission [5] highlight the critical role oceans must play in sustainable and equitable food futures.

Transforming food systems demands social change and innovation in how food is produced, traded and consumed. ‘Social innovation’ (SI) involves new products and services, collaborative ways of working, novel governance arrangements and business models designed to generate systemic change, delivered through partnerships, often place‐based, to respond to social, environmental and health needs [6]. Critically, SI is embedded within, and dependent on, the wider policy ecosystem since policies can enable or impede development, diffusion and scaling [6, 7, 8]. Yet, the policy mix shaping food systems is fragmented across multiple sectors, policy levels and actors including government, private and third sector organisations [9, 10, 11, 12, 13]. Calls for greater integration and coherence across this complex landscape are longstanding [13, 14]. Recognising that boundaries between formal policies and practitioner‐led initiatives are often blurred, we adopt the label ‘policy and practice’ in this paper.

These calls align with a broader shift towards systems thinking within food systems policy and research [15]. ‘Systems thinking’ [16] offers a means of moving beyond reductionist or linear approaches [17] to confront ‘wicked’ issues and embrace complexity [18]. Systems thinking is valuable to a wide range of fields including public health policy, research and practice [19]. While this way of thinking is proposed, its implementation by practitioners, including those working in nutrition/health, requires a professional cultural shift, for example, more consistent integration into educational curricula is needed to overcome perceived resistance to change [20] in those who practice using traditional/reductionist approaches. This shift requires stronger collaborative leadership and advocacy skills [21].

This paper draws together the themes of food system transformation, social innovation (SI) and the need for more systemic food policy and practice actions, using the case of the UK's Plymouth Fish Finger (PFF) as an exemplar. The PFF is a collaborative SI case study [22] which has pioneered community‐centred blue food system change. It utilises under‐valued locally procured fish species (supporting fisher livelihoods), co‐creates with schools a new product, and diverts this into the school meal system (improving fish intake in children) [23]. Part of the FoodSEqual research project [24, 25], PFF champions ‘co‐production’ approaches [26] [defined as ‘a collaborative way of working which emphasises the exchange of diverse forms of knowledge in an equal partnership for equal benefits’] [27] and multi‐stakeholder collaboration [28] to achieve multiple (potential) impacts, including: (i) innovating a novel seafood product (culturally‐acceptable and ‘sustainable’ fish finger); (ii) ‘disrupting’ traditional fish supply chains, by localising processes; and (iii) informing policy, practice and ‘blue food system’ discourses.

Our study reports on this exemplar of SI good practice to elicit how the role of the (policy and practice) system around the PFF can enable and inhibit its realisation, to inform and optimise SI practices for wider (blue) food system transformation. The paper is based on the following key questions:

(1) What policy and practice system is needed to support the PFF innovation?

(a) How can visual mapping methods illuminate system complexity and identify leverage points for intervention?
(b) What approaches can be used to optimise engagement with policy/practice advocates to elicit expertise to inform impact?

## 2 Methods

### 2.1 Design

An expert elicitation process [29] was combined with subsequent (online) group model building (GMB) [30, 31] to co‐create a systems map—a ‘Causal Loop Diagram’ (CLD)—to visually understand and analyse the policy and practice ecosystem around the PFF initiative.

Expert elicitation has a long history of use across disciplines including conservation [32], environment [33], policy [34] and, more recently, food systems [35]. When empirical data are limited, this process enables experts to come together to discuss complex topics to inform the evidence base [36]. This method has inherent biases, due to results not being easily reproducible [37], but a structured approach can mitigate such limitations. Within expert elicitation, the number of experts chosen varies, depending on the depth/range required, with researchers usually opting for 5–20 experts [38].

Using experts to map systems is becoming more commonplace [39], yet methods are still evolving [40]. GMB was chosen as a well‐established approach whereby system actors (i.e., expert stakeholders from civil society, academia, policymaking and business) share their views about problems to create collective understandings of the system [41] to uncover its causal structures and outcomes [31]. These can then be visually produced into a CLD map, which facilitates identification of leverage points, inhibitors and enablers, to then target actions and interventions for systemic changes [30].

### 2.2 Recruitment

Purposive sampling [42] was used to recruit experts to attend two workshops (Table 1). Already established networks—an evolving PFF ‘Community of Practice’ (comprising over 25 stakeholders)—were used for this purpose. The sample aimed for diverse representation of fish/seafood experts across the system (fishing, processing and distribution, catering, sustainability, standards, certification and education). All were anonymised to maintain ethical integrity.

**Table 1:** Expert workshop participants by sector (anonymised).

Sector representation	Workshop 1	Workshop 2
Social enterprise (supports fishing community) × 2	Y (×2)	Y (×2)
Social enterprise (interest in fishers)		Y (×2)
Local authority (economic development)	y	y
Local authority (catering)	y	y
Local authority (Catering)		y
Fishing industry/UK public body supporting seafood industry	y	y
Marine expert (academic)	y	
Marine biologist (academic)		y
Journalist (interest in fish)	y	
International non‐profit organisation that promotes sustainable fishing and protects oceans by certifying and labelling	Y (follow‐up online meeting)	
UK‐based charity that works to protect seas, shores and wildlife	y	
Policy Institute (Seafood expert)	y	
National government department	y	
Fisher	y	
	*N* = 11 (+1)	*N* = 9

### 2.3 Workshop Delivery

**Figure 1:**
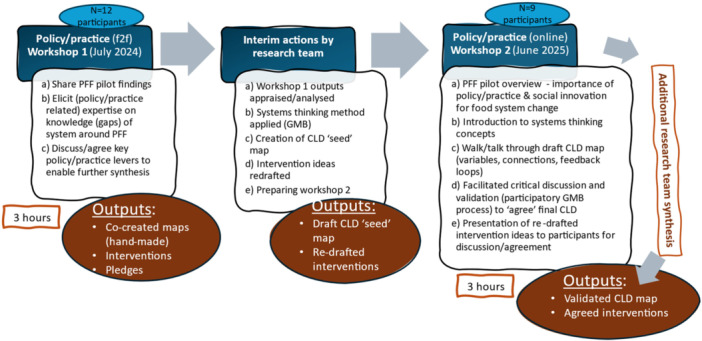
Workshop delivery process for PFF systems mapping.

Two expert elicitation workshops were run, a year apart, with progressive interim actions by the research team implemented between workshops. The research team are expert facilitators with extensive experience of participatory workshops. See Figure 1.

#### 2.3.1 Policy and Practice Workshop 1 (July 2024)

Based on extensive experience of delivering participatory workshops for optimal engagement [43], creative, interactive ‘visual mapping’ techniques were used, inspired by a food systems mapping tool created by Sustainable Food Places [44] and drawing on the approach used by White et al. [45]. This enabled identification of: (i) where the PFF interacted with local and national policy/practice elements and (ii) where change might be possible. Participants (experts) were asked to ‘map’ the system around the PFF, using colour‐coded post‐it notes with ‘inner circle’ (yellow) representing local policies/practices and ‘outer circle’ (orange) representing national policies/practices. Then pink post‐its were added to identify what interventions were needed and where, for example, changes to local or national policy or practice, to make the PFF innovation viable. There are different approaches to identifying the policies which are relevant to a social innovation field, which can be broadly understood as either top–down and/or bottom–up delineation [46]. We opted for a ‘bottom–up’ real‐world approach to policy identification, asking field actors to delineate policies impacting on the SI field of the PFF, whether intentionally targeting SI or not. This is because there are many policies—for example, public sector procurement policy, education policy—which are not directed at SI but impact on its success [46]. We were not looking to identify *SI policies* which target the PFF. Such a ‘top–down’ approach would have delineated SI policies—any policy which is explicitly about social innovation, such as incubation or acceleration support—as relevant. This would have been too limited given the breadth of the PFF social innovation field [46]. It was also important that the relevant policies and practice were identified through participant expertise. Intervention ideas were harvested and then, subject to feedback from participants on their relevance and feasibility, prioritised through a basic voting exercise (using sticky dots). To align with good ‘co‐production’ practice [26] participants were also guided through a ‘pledge‐making process’ which involved individually recording a statement on a post card. This enabled participant agency to express commitment to the project and informed observations.

**Figure 2:**
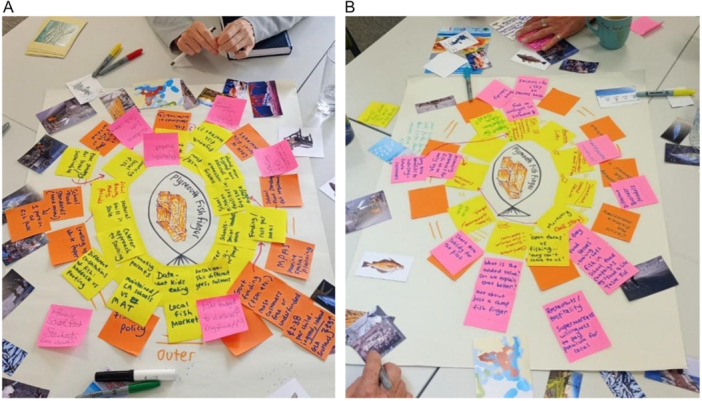
PFF mapping outputs from Workshop 1. (A, B) Maps were organised concentrically, with local policy/practice elements (yellow post‐its), national elements (orange post‐its) and changes required (pink post‐its).

One participant, who could not attend in person, was engaged separately through an online version of the process. Workshops were not audio‐recorded, instead research team members observed closely and took high level notes, whilst preserving anonymity. A workshop summary was sent to all participants which included photographs of maps (Figure 2A,B) and outlined interventions and pledges.

#### 2.3.2 Interim Actions by Research Team

Maps from Workshop 1 were critically appraised by the research team (P. C. and P. K.), alongside participant intervention ideas and pledges. Some basic codes and themes were extracted manually to synthesise data, informed by Braun and Clark [47]. Given the complexities identified from these preliminary analyses, it was decided to apply systems thinking methodology using GMB principles and produce a ‘CLD’ [31], based on the data extracted and other observational high‐level notes. A draft CLD was co‐created by the research team (P. C., P. K. and I. Y. C.‐U.) during several online meetings, by systematically translating the qualitative information into CLD core elements (i.e., variables, connections and feedback loops) and themes, identified by the themed colours in the map, to exemplify the systems complexities and visually inform the design and delivery of Workshop 2. Concurrently, the research team organised and re‐drafted the intervention ideas according to their corresponding location within the draft CLD. A follow‐up email was then sent to participants, asking them to attend a second workshop. At this point the summary from Workshop 1 was attached, plus the preliminary draft CLD with explanatory notes.

#### 2.3.3 Policy and Practice Workshop 2 (June 2025)

Policy and practice experts were brought together again (online) to review and refine outputs from Workshop 1. The draft CLD was presented as a ‘*seed map*’ [48] to support a GMB process, which was chosen because it enables stakeholders to collectively validate system structure, integrate diverse expertise and build shared understanding of complex food system dynamics [31]. The CLD was developed as a qualitative structural model, focusing on stakeholder‐perceived causal relationships, feedback structures and leverage points for action rather than modelling behaviour over time.

In this study, the decision was taken not to include an explicit reference mode elicitation step—to identify, define and graph the historical or expected behaviour of key variables over time. This was due to the online delivery format, time constraints and the study's objective to develop a qualitative CLD rather than a quantitative system dynamics model [41]. Instead, system boundaries were defined through a shared problem framing and iterative stakeholder discussion, centring on factors influencing whether locally caught fish could remain within the local supply chain and enter the school meal system.

As a result, the workshop focused on identifying perceived causal relationships and feedback structures underlying the issue. Based on this, participants sense‐checked variables and connections and identified missing elements. The workshop then engaged participants in reviewing a list of potential interventions which could be introduced, to remove barriers and enable the PFF as a social innovation. Prior to the second workshop, interventions identified in Workshop 1 (pink post‐its) were collated in a table (Table 2), and assigned to the thematic area of the CLD they targeted, with each thematic area colour‐coded with the same colours used in the CLD. Interventions were assigned to themes based on their location on the Workshop 1 maps and through iterative consideration of the CLD and the list of interventions. This followed the approach used by White et al. [45] to organise intervention ideas according to the system elements or ‘leverage points’ within a CLD. Additional considerations related to the introduction of each intervention, which were raised by participants, were also included in the table.

**Table 2:** PFF interventions as identified during expert elicitation and systems mapping processes.

System map theme/area	System element(s) addressed	Proposed intervention	Rationale	Associated governing entity, or potential actor to deliver	Considerations raised by participants
Economic viability	Capacity for PFF production at scale Funding for equipment/mechanisation, processing & space Organising financial capacity for PFF	Short‐term subsidy for extension of PFF pilot (subsidy/funding for key PFF players) to enable move from hand production to commercial footing Funding is required for: 1. Project management/leadership and ongoing collaboration activities (given cessation of main FoodSEqual research funding for research team) 2. Coordination of the PFF supply chain 3. Capital investment in equipment (PFF machine for production and associated overheads)	Collaboration of the community of practice, and coordination of the supply chain require (monetary and in‐kind) resources to be maintained, and to produce proof of concept on a smaller scale and demonstrate holistic economic benefits (e.g., to the local economy) Funding for the creation of a local processing facility will alleviate bottlenecks of processing capacity and PFF supply	Various options available, including: Grant/Philanthropic Funding National or Local Government ‘Innovation’ or ‘Enterprise’ Funding Private Sector Funding Other ‘investment’ options	Trust is an essential ingredient in the PFF SI, and maintaining relationships of trust requires active support, transparency and clear lines of communication. There have been challenges with sourcing suitable machinery for up‐scale. Identified options for processing are for much larger quantities, and therefore very expensive.
	Budget for school meals Affordability of PFF	Increased budget for (free) school meals	Would allow increase in amount feasible to pay per PFF by school caterer, and reduce financial friction in the PFF supply chain	National Government	Current budget per meal £2.61 (recently increased from £2.58)—makes incorporating PFF into menus challenging, as competing with cheaper industrially processed lower‐quality products
	Viability of livelihoods of small boat fishers	Grants (and incentives) for fishing community/industry	Provide support for fishers who are needed as primary suppliers of PFF species, but whose current livelihoods are precarious	National Government; Local Government Non‐Government organisations, charities, etc.	Support for the UK fishing industry in the UK tends to be very prescriptive, for example, for particular projects or equipment, and not more general
Demand generation	Knowledge, awareness and preference for different species Fish literacy of school students Education about the value of fish (health, environment, local economy)	Fish education in schools: children‐focused	Early and repeated exposure to fish education alongside taste testing can shift norms and demand for schools, chefs, social enterprises.	School leadership (but will require resource/support to deliver) Current organisations participants thought were delivering similar: Soil Association Food4life; FishHeroes; Taste Ed; Seafish; Jamie Oliver Could be covered by ongoing (research pilot) funding in short‐term	Should be an ongoing (embedded) programme not just one‐off exercise Matching local fishers with schools would be impactful There are challenges with educational visits to fishing quays and processing facilities (compared to, for example, school farm visits). Could relate to safety and/or lack of access
	School preference for other food not local fish	Procurement policy/strategy, embedding the principles of prioritising local suppliers including Plymouth small boat fishers, and prioritising other objectives than cost	Would support the procurement of PFF, benefit the local economy and reduce friction in the PFF supply chain	New policy could be introduced at multiple levels: Organisational (school) Local Government (Procurement principles for local anchor institutions) National Government—as part of procurement policy	Requires a matrix approach to principles, which encompasses both environmental and social impacts of buying local/the PFF It is a complicated story to communicate in relation to fish sustainability and the complexities of product (type of fish) and production process (method of fishing), which acts as a barrier to change
	School cook skills, knowledge of local fish PFF literacy of chefs Leadership from schools to support PFF Education about the value of fish (health, environment, local economy)	Education/training: School ‐caterers	Preference for ‘Big 5’ fish species means caterers may not be familiar with other/local under‐utilised species, which acts as a barrier to procuring and encouraging PFF consumption	Could be integral part of a new procurement policy and thus introduced at multiple levels as per above. Focus on under‐utilised species could be embedded into professional catering training.	As above, it is a complicated story to communicate in relation to fish sustainability and the complexities of product (type of fish) and knowledge of preparation/cooking method. There are unintended consequences of making fish so popular that it ends up being over exploited (even underutilised species). Needs exploration to establish if this is a practical or perceived barrier. Most different fish species can be cooked in a similar way if they are already at least partly prepared, for example, filleted.
		Education/training: Teachers	Education about the value of fish (to health, environment, local economy) requires teachers to be knowledgeable, and can support PFF consumption	Teacher training institutions could play an active role in enabling fish literacy Could be included as part of national curriculum, by National Government	Must be embedded as part of the education curriculum, otherwise it may not be prioritised. It is a complicated story to communicate in relation to fish sustainability and the complexities of product (type of fish) and preparation/production process (method of fishing), which acts as a barrier.
	Demand for PFF from online retailer Subsidised cost of PFF for schools	New local fish sourcing strategy: supermarkets and food service/hospitality (non‐school buyers)	Purchase of PFF as ‘premium product’ from larger businesses (which can afford to pay more) could offer a ‘Robin Hood approach’, providing a cross subsidy to public‐sector buyers.	Private sector businesses	Supermarkets, due to their complex logistics and long supply chains, do not tend to buy local, which represents a barrier.
	Knowledge, awareness and preference for different species Education about the value of fish (health, environment, local economy) Student preference for fish	Promotion to parents	Parents influence the meal choices made by children, and consumption of fish at home can support taste preferences, including increasing preference for fish at an early age Early and repeated exposure to fish education and taste testing can shift norms and demand for families, schools, chefs, social enterprises.	Whole school community	It is a complicated story to communicate in relation to fish sustainability and the complexities of product (type of fish) and preparation/cooking/production process (method of fishing), which acts as a barrier to change for family/community Could involve a practical element, for example, serving PFF at parent teacher nights and other events
	Knowledge, awareness and preference for different species Education about the value of fish (to health, environment, local economy)	Marketing and communications—‘selling the story’ of the PFF and fish more generally	Early and repeated exposure to fish education and taste testing can shift norms and demand for schools, chefs, social enterprises.	Public sector institutions, private sector businesses, Local and National Government, civil society; non‐government organisations	It is a complicated story to communicate in relation to fish sustainability and the complexities of product (type of fish) and preparation/cooking/production process (method of fishing), which acts as a barrier to change
	Fish intake in schools required to comply with school food standards	Amendment to school food standards	Current levels of consumption in schools are (negatively) influenced by the requirement to only serve fish once per week	National Government	Requirement of one serving legitimises inclusion of fish on menus but acts as a ceiling for procurers/caterers which inhibits the market for the PFF School Food Standards currently being reviewed so policy window currently open
Supply chain constraints	Public and business access to local fishing industry (e.g., Access to local fish market) Supply of local fish (Access to local fish and availability of underutilised (low value) species)	Re‐opening of Plymouth Fish Market, supported through removal of business rates and protection of market via planning policy, to aid economic viability of key part of the local food infrastructure	Processing currently takes place outside Plymouth, which adds costs and other logistical challenges to the PFF supply chain	Commercial entity would be needed to re‐start a market Potential role for Local Authority	Landlords have control of the market Visioning activities are currently underway to re‐develop the fish market space The lack of a market has led to fewer boats landing in Plymouth which means reduced access to direct sales (and potential PFF production)
		Establish a direct purchasing arrangement from vessels landing in Plymouth, or failing that from vessels landing to other ports	Direct access to fish for processing would circumvent the market and transport costs and should therefore be financially beneficial for both PFF team and fishers Buying from other ports may be necessary to increase supply/meet demand but viability depends on price/costs	PFF team would be the ‘actor’ initiating the purchase Support from wider community of practice?	Not yet clear if viable to drive fish from other geographical (coastal communities) to PFF facility for processing.
Unintended negative consequences	Unintended consequences of incentivising under‐utilised species Overshooting local fish availability Big 5 purchasing to meet demand for fish	Monitoring and Evaluation: A system for measuring ecological and social impacts PFF project should collect data on fish procured for processing. This would link into and enhance existing knowledge on stocks used to ensure sustainability	Will help ensure long‐term resilience and allow for system adaptation when required, for example, to ensure under‐utilised fish do not become over‐fished.	National government bodies responsible for marine data (e.g., Marine Management organisation MMO) Non‐government organisations that are responsible for certification and standards (e.g., Marine Conservation Society Good Fish Guide; International Union for Conservation of Nature IUCN) The PFF project team could initiate data collection as part of the project (with fishers involved) Scientists and researchers (within local research institutions) have expertise to support	Lack of availability (and policing) of data means level of monitoring required is limited. Fishers could help collect data but would be preferable to employ someone or find a way of doing it as part of processing. The hope would be that there is funding available for this. Reality is that knowledge of fish stock status is often poor for underutilised species (often called data deficient species as a result). Caution is needed, therefore, to learn more about their stocks before encouraging too much fishing pressure on them

*Note:* Funding for equipment/mechanisation, processing and space leads to Capacity for PFF production at scale/Processing financial capacity for PFF. At the time of writing this research paper, the research team received confirmation of funding to purchase the machinery needed to mechanise PFF production, which is a key element in feedback loop B, Mechanisation‐Cost loop.

The final part of Workshop 2 involved asking participants to provide formal feedback on the mapping processes and their usefulness. With consent, discussions were recorded and transcribed (using Zoom software). The research team then reviewed inputs and revised the CLD, ensuring it accurately reflected stakeholder knowledge/expertise and the dynamic system surrounding the PFF initiative.

### 2.4 Ethics

This project was approved (Ref: 5208) by the Faculty of Health Research Ethics & Integrity Committee at the University of Plymouth.

## 3 Findings

### 3.1 Outputs From the Expert Elicitation Process

a. Preliminary maps (Workshop 1) were crafted by hand by expert participants (Figure 2A,B).

b. The system around the Plymouth Fish Finger (Workshop 2).

**Figure 3:**
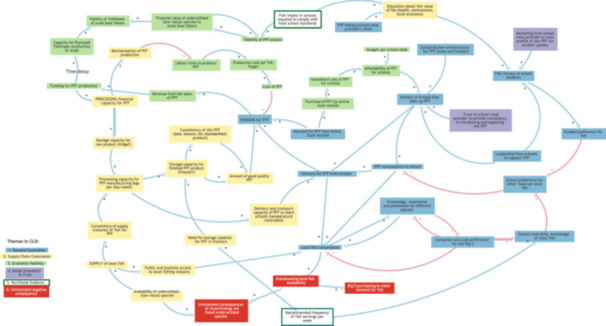
Overview of the system map. This diagram presents the stakeholder‐generated causal loop diagram (CLD) illustrating the social, economic, environmental and logistical factors influencing whether locally caught fish can be retained within the Plymouth supply chain and incorporated into school meals. Variables are grouped into six colour‐coded themes: Demand Generation, Supply Chain Constraints, Economic Viability, Social Innovation and Trust, Nutritional Guidance and Unintended Negative Consequences. Arrows indicate causal influences, with ‘+’ denoting same‐direction change and ‘–’ denoting opposite‐direction change. The map reflects collective insights from policy, practice and industry participants and provides an integrated representation of the system dynamics surrounding the PFF initiative. Adds to/ same direction. Subtracts from/opposite direction.

The systemic dynamics underpinning the initiative (how elements are interconnected and how these relationships give rise to reinforcing and balancing feedback dynamics) are illustrated in the CLD in Figure 3 (see also KUMU link here https://tinyurl.com/32hayx68). The map visualises participant perspectives on how social, economic and supply chain dynamics interact within the PFF system to influence the SI's capacity to keep locally procured fish within the local supply chain so that it can then enter school meal provision.

The CLD contains 49 elements connected by 130 causal links and 5 feedback loops. The six colour‐coded themes represent distinct but interconnected domains of influence, within which reinforcing and balancing feedback dynamics shape system behaviour. The findings describe perceived system dynamics and constraints as articulated by stakeholders, rather than observed temporal trends, and are presented by theme, with feedback loops embedded to illustrate how system dynamics emerge across the PFF system.

Creating meaningful demand among consumers, schools and catering services was identified as a foundational condition for the PFF initiative (Figure 3, right‐hand side). Stakeholders emphasised the importance of building awareness and literacy around local fish through educational activities, marketing and leadership within schools to normalise the inclusion of the PFF in school menus.

Theme 1 Demand Generation (blue)

**Figure 4:**
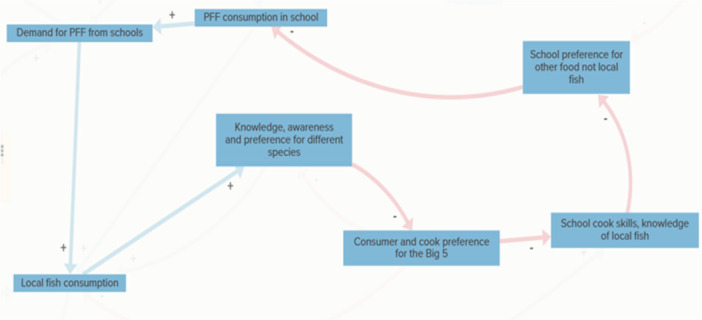
Demand–viability loop. This feedback loop is heavily influenced by an increase in fish literacy, marketing and school leadership which creates an awareness and enthusiasm for the PFF and is critical in maintaining the demand‐viability feedback loop. As demand grows, PFF consumption rises, completing a cycle in which educational, cultural and culinary readiness work together to support the viability of the PFF in school settings.

Within this theme, the demand–viability loop (Figure 4) illustrates how increased uptake of the PFF in schools enhances local fish consumption more broadly, strengthening knowledge, awareness and preference for different species. This reduces reliance on the commonly consumed ‘Big 5’ species and increases openness to locally available alternatives. Increased confidence among school chefs and reduced preference for non‐local foods further reinforce demand. However, financial limitations in school meal budgets and entrenched preferences for familiar foods act as constraints, moderating the strength of this demand‐driven dynamic.

Theme 2 Supply Chain Constraints (yellow)

The PFF requires a reliable and consistent locally procured fish supply, yet stakeholders identified significant logistical and infrastructural barriers (Figure 3, left‐hand side). Limited processing capacity, insufficient fridge and freezer storage, and constrained temperature‐controlled delivery affect both the volume and consistency of supply, as well as product standardisation in terms of taste and texture.

**Figure 5:**
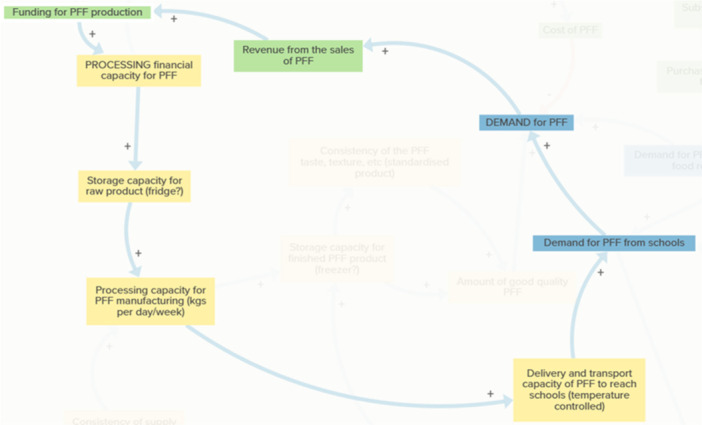
Delivery and infrastructure capacity loop. Logistical and infrastructural capacity shape the feasibility of supplying schools with the PFF. Rising demand increases the need for reliable, temperature‐controlled delivery and transport, as well as sufficient processing and storage capacity. If these infrastructural elements are insufficient, producers may be unable to meet school expectations for timeliness or product condition. Reduced ability to supply good‐quality PFF can lead schools to reduce ordering, diminishing revenue streams needed for infrastructure improvements. In this way, infrastructural gaps create a balancing effect that slows or limits the growth of the PFF initiative even when interest from schools is strong.

Two feedback dynamics are embedded within this theme. The delivery and infrastructure capacity loop (Figure 5) demonstrates how rising demand increases pressure on transport, storage and processing infrastructure. Where capacity is insufficient, producers may struggle to meet school expectations for delivery and product condition, reducing the availability of good‐quality PFF and leading schools to scale back orders. This, in turn, limits revenue available for infrastructure investment, slowing system expansion.

**Figure 6:**
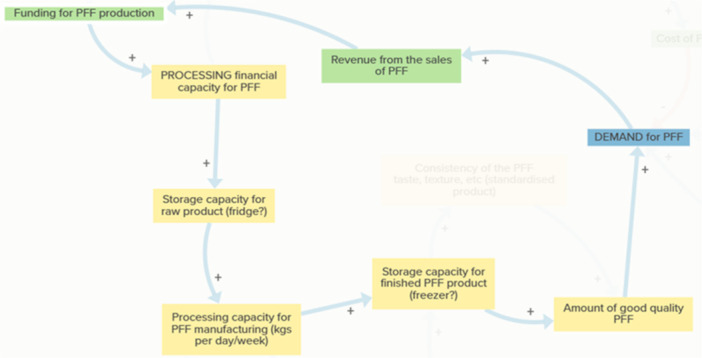
Processing capacity loop. Investment in processing infrastructure supports PFF availability and market stability. Processing capacity needs sufficient storage for raw materials and robust financial capacity for processing to enable producers to increase the quantity and reliability of PFF production. Higher volumes of good‐quality PFF increase demand from schools, which generates additional revenue from sales. This revenue reinforces the financial base needed to maintain or expand processing capacity. Thus, improvements in processing infrastructure and financial reinvestment work together to support growth in local fish utilisation.

In parallel, the Processing Capacity Loop (Figure 6) shows how sufficient storage and financial capacity enable increased production of good‐quality PFF, supporting demand and revenue generation. However, small‐scale fishers and processors often lack the capital and coordination needed to respond quickly to increased demand, reinforcing supply‐side constraints.

Theme 3 Economic Viability (green)

Economic viability (Figure 3, top left) reflects the delicate balance needed between maintaining affordability for schools and ensuring fair income for small‐scale fishers and processors. Labour costs, production expenses and market price pressures are challenges.

**Figure 7:**
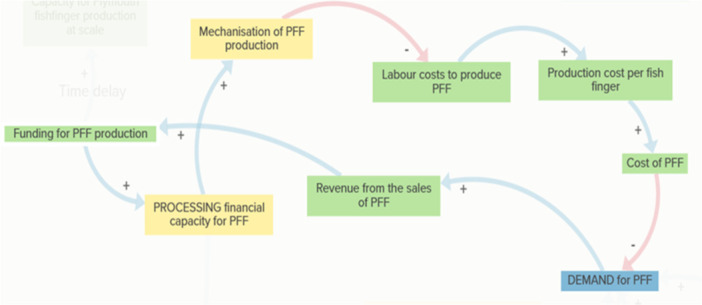
Mechanisation–cost loop. Technological improvements in production interact with economic constraints faced by schools. Increased mechanisation of PFF production reduces labour inputs, lowering production costs. This cost reduction typically improves affordability for schools, contributing to higher demand and increased sales revenue. If production costs or final prices increase beyond school budget limits, demand is reduced. This loop therefore acts as a self‐regulating mechanism: while mechanisation initially improves affordability and uptake, financial constraints within school food budgets ultimately stabilise or limit expansion. Currently, the PFF pilot shows that when mechanisation is possible, there will be enough production to match school demand locally and further scale‐up might not be necessary, keeping operational costs relatively stable.

The Mechanisation–Cost Loop (Figure 7) captures how technological improvements in production reduce labour inputs and lower production costs, improving affordability for schools and supporting increased uptake. However, this loop also acts as a stabilising mechanism: if production costs or final prices increase beyond school budget limits, demand is reduced. Stakeholders noted that within the current PFF pilot, mechanisation enables sufficient production to meet local school demand without requiring further scale‐up, helping to keep operational costs relatively stable.

Stakeholders noted that scaling production would require capital investment, potentially increasing costs. Subsidised pricing or bulk purchasing by large buyers (Figure 3, top centre) were seen as potential stabilisers, but with risks of market distortion and pressure on fish stocks if demand increases without appropriate safeguards.

Theme 4 Social Innovation and Trust (purple)

Although represented by only two elements in the map, trust emerged as a critical enabling condition with effects across the system (Figure 3). Trust between schools, caterers and local producers was seen as essential for maintaining confidence in product consistency and delivery reliability.

**Figure 8:**
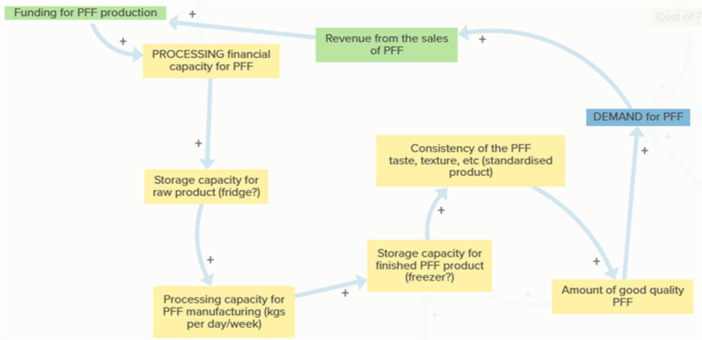
Consistency–quality loop. Sustained demand is dependent on product quality. When producers have adequate processing capacity and finished‐product storage, they are better able to deliver PFF that meet consistent standards of taste, texture and appearance. High product consistency reinforces confidence among school caterers and decision‐makers, strengthening demand for the PFF. Increased demand generates revenue that can be reinvested into maintaining or improving production processes, preserving the quality and consistency that schools value. This loop underscores that the reliability of the PFF as a food service product is not only a matter of processing technique but also a function of coordinated storage, capacity and financial reinvestment.

Trust is embedded most clearly within the Consistency–Quality Loop (Figure 8). Adequate processing and finished‐product storage enable consistent PFF quality, reinforcing confidence among school caterers and decision‐makers. Increased confidence strengthens demand and participation in the PFF, generating revenue that can be reinvested into maintaining quality. Conversely, disruptions in supply or inconsistency in product quality can quickly erode trust, dampening demand and slowing innovation uptake. This theme underscores that social innovation depends as much on relationships and reputation as on technical or economic performance.

Theme 5 Nutritional Guidance (green box)

The Nutritional Guidance theme situates the PFF initiative within the broader system of dietary policy (Figure 3, bottom and top‐middle). National school food standards and recommended weekly fish servings influence both the frequency and type of fish served in schools. Currently, standards recommend only one fish serving per week and do not require local sourcing, while school food procurement budgets remain low. An enabler for school's uptake would be guidelines supporting purchase of local innovative products that mandate twice weekly fish servings, and school meal budgets aligned to the costs of this.

Educational activities linking the health, environmental and economic benefits of local fish are viewed as critical to sustaining institutional and community support. This theme highlights that nutritional policies currently act as a brake/inhibitor, but disruption or change to these might turn them into an enabler.

Theme 6 Unintended Negative Consequences (red)

Up‐scaling the PFF initiative has potential unintended outcomes. Increased demand for local under‐utilised species could inadvertently encourage the harvesting of red‐listed or vulnerable fish species, undermining sustainability objectives (Figure 3, bottom central). Excessive focus on the PFF as a single product could divert attention from broader food system improvements or strain existing infrastructure (e.g., production output, storage, distribution). These dynamics introduce ecological and systemic constraints that limit expansion and highlight the need for adaptive governance and ecological safeguards, and ongoing monitoring to ensure that local sourcing strategies do not compromise marine biodiversity or long‐term system resilience.

### 3.2 The Dynamics of the Feedback Loops Working Together

Across all themes, the embedded feedback loops (Figures 4, 5, 6, 7, 8) demonstrate how the PFF system is shaped by reinforcing dynamics that build momentum through demand generation, processing capacity, quality and trust, alongside balancing dynamics related to affordability, infrastructure, nutritional policy and ecological limits. Integrating feedback loops within thematic domains provides insight into where targeted policy and practice interventions may strengthen enablers, mitigate constraints and support the sustainable integration of local fish into school meals.

c. Discussion from participants on utility of systems map approach

Along with input into the content of the CLD, participant comments about its utility were also noted. The importance of the narrative supporting the map (including key concepts) was acknowledged. One participant critiqued the boundaries and limitations of the map and how it potentially over‐simplifies complexities within the system. Terminology was raised as a potential issue and the importance of ensuring terms are understood consistently across different sectors/disciplines.

### 3.3 Interventions Which Could Be Used to Support Delivery of the PFF

Based on the CLD, several potential interventions were identified (Table 2). Most interventions proposed by participants related to either the need for demand generation from institutions and citizens, including school children and families, or to supporting economic viability of the supply chain. Interventions are organised according to the six CLD themes and the respective system elements addressed (informed by a validated approach [44]). Participant input on potential responsible agent(s) for this aspect of the SI field, and other considerations raised during workshop discussions and the wider PFF project, are also presented for each intervention proposal. Participants offered important reflections about intervention ideas harvested in Workshop 1, which were presented back during the second workshop. This illustrates the need for continued co‐production of intervention ideas with relevant stakeholders, ensuring proposed actions are shaped using the exchange of diverse forms of knowledge, to ensure their ongoing effectiveness/impact and to avoiding, where possible, unintended consequences from introducing interventions into one part of the system, which have ripple effects elsewhere.

## 4 Discussion

This study used the example of the PFF to demonstrate how the complexities around a blue food system social innovation can be understood. Using an expert elicitation process, combined with subsequent (online) GMB a ‘CLD’ (systems map) was co‐created and validated by workshop participants, along with a set of intervention ideas. We believe our processes with the PFF can offer important introductory understanding that can inform future research and mapping analyses. Findings are presently critiqued.

### 4.1 Mapping Complex System(s)

The CLD tells the story of how a locally co‐produced PFF has the potential to become an ‘social innovation game changer’ [49]. On deeper consideration of the PFF system, several key facets are uncovered relating to demand, supply, economic viability and social innovation. The feedback loops identified in the CLD highlight the dynamic conditions that influence whether locally procured fish can be sustainably integrated into school meal provision. Reinforcing mechanisms, such as those linking demand, processing capacity and product consistency, demonstrate how educational engagement [50] and fish literacy [51], reliable supply and high‐quality production can collectively enable the adoption of this social innovation. Concurrently, balancing mechanisms related to affordability and infrastructure reveal practical limits that constrain delivery of more sustainable food and scale‐up, within current public food procurement [52] and supply chain/logistical systems [53]. These dynamics suggest that policy and practice interventions must be introduced to create the enabling conditions that embed demand and viability, whilst simultaneously addressing the structural constraints that dampen system performance. These interventions are interdependent and reinforcing, as discussed further below.

Our mapping processes support systems thinking by engaging stakeholders in conceptualising and building consensus around complex challenges [54], in this case relating to the food system. The GMB process enabled visualisation of how different parts of the system relate to one another [55], a process which has been previously tested for online use [30]. The resulting map represents the complexity of the PFF system in visual format, making it more accessible. The map represents a ‘boundary object’ [56], which can allow shared exploration of relevant policy and practice, within the diverse community of practice of the PFF's SI field.

Our processes also offer some degree of novelty. Within food systems research, participatory systems mapping is beginning to receive more attention. Kiraly et al. [57] assessed the participatory potential of systems mapping suggesting that the more people involved in co‐constructing processes, the more detailed and nuanced a picture can emerge of the relevant multifaceted social reality [58]. Also important is the knowledge that engaging with diverse stakeholders can catalyse practitioners and researchers' self‐reflection and reflexivity [59, 60], which within the complex space of food system transformation research, is core to advancing and scaling participatory and transdisciplinary methodological developments [26, 61]. We believe our processes, positive engagement from expert blue food system stakeholders and resulting visual CLD output reinforce the utility of participatory systems mapping.

### 4.2 Expert Elicitation Informs Deeper Understanding of Systems Thinking

Expert elicitation provided our study with a range of perspectives [36] and disciplines to feed into our GMB process [31]. This supports the utility of GMB to effectively engage participants [62] including in an online context [30]. Based on our observations and outputs, participants (experts) engaged well with the CLD map and the ensuing discourses between them, illustrated how their expertise can inform a deeper understanding of systems dynamics. For example, tensions were raised in relation to the balance between production versus demand for the PFF and this was flagged as an ‘unintended consequence’ [Figure 3: availability of underutilised (low value) species] in that a drive for demand might lead to risk of over‐exploitation of the fish species [63]. Affordability and cost‐effectiveness aspects were also discussed [Figure 3: funding for PFF production; subsidised cost of PFF for schools]. There is known fish‐price volatility (globally and locally) which can negatively affect small‐scale fish traders [64], so their cause needs support and investment via grants and incentives (Table 2). Although beyond the scope of this study, examples of existing policy measures, such as fiscal incentives for production or dynamic procurement processes merit further exploration for application to PFF. Similarly, lack of resource is commonly seen as a challenge for SI projects, which are often precarious, relying on grants and/or diversifying with multiple funding sources [6]. Finally, participants identified that the map over‐simplified the PFF system. We acknowledge this point; indeed, the food system is a complex network of interconnected actors and activities [65, 66] and this re‐emphasises the critical importance of taking a systems perspective [16]. We also fully justify our visual map as a powerful vehicle to acknowledge and navigate/embrace this complexity. Our systematic processes have enabled participatory integration of discourses that can both facilitate systems thinking and consolidate collaboration [28] to improve future blue food systems policy‐ and practice‐related action research.

### 4.3 Identifying Interventions for Future Action

Across all six CLD themes, stakeholders described the PFF initiative as an important example of embedding social innovation within a local (blue) food system. Success, however, depends on the alignment of multiple policy domains, including education, nutrition, fisheries and procurement, supported by strong skills and practices of trust and communication between institutions and communities. There are obvious tensions here between the inherent social values within SI practice [6] and how scale‐up towards commercialisation, which might be essential for viability, may be seen as compromising such values [67]. The analysis suggests that interventions aimed at improving local fish integration in school meals must balance economic feasibility, social innovation and trust, and ecological responsibility. Strong leadership, system‐wide coordination [1], continuous stakeholder engagement [28] and transparent evaluation processes [68] were consistently identified as effective ways to maintain momentum and adapt to changing conditions. However, this is held in tension with the identified precarious funding ‘cliff edges’ [6] whereby leadership responsibilities may shift or be lost over time.

We propose two important advantages of using systems mapping to understand enablers of a social innovation. First, it offers a way of identifying the package (i.e., series of identified interventions) of required complimentary policy and practice changes. Policy packaging—sometimes referred to as bundling—is an established concept within policy sciences and becoming an increasing focus within food system transformation discourse [52, 69, 70]. But practical demonstration of its design and delivery in the real‐world is rare. The PFF illustrates how failure to deliver interventions in concert—covering both stimulation of demand and facilitation of supply—will mean system dynamics undermine the success of the social innovation. In particular, it illustrates how educational interventions, while crucial for addressing demand, are insufficient alone and require more structural and supply‐focussed action [71, 72, 73] within a values‐driven SI leadership context. This, in turn, requires coordination between actors across multiple food system policies and practices, which is unlikely to happen without formal mechanisms, such as project coordination, in place [1, 11, 52]. Second, the map can be used to observe and avoid unintended consequences by identifying the ripple effects of one intervention across the wider system [74]. A future research recommendation is to use the CLD as a ‘living tool’ to further analyse the feedback loops presented to judge/evaluate how the system adapts and changes once interventions are put in place.

### 4.4 Strengths and Limitations

This study is the first of its kind to combine expert elicitation with systems thinking in a blue food system context, effectively appraising the system around this exemplar PFF social innovation project by co‐creating a visual systems map. The study has strengths relating to the diverse stakeholders who engaged with the workshops representing the blue food system [28]; a robust methodological framework underpinning its processes, informed by systems mapping, GMB [31], expert elicitation [30] and alignment with the principles of systems‐based social innovation [6].

There are, however, inherent limitations in this study. The small purposive sample may have led to sampling bias [75], with some voices not being represented. For example, a nutrition professional as a participant would have served to enhance this thematic element. The short timeframe to conduct the research meant processes felt rushed: with more time, creative participatory approaches could have been further developed [26]. Not including an explicit reference mode departs from standard GMB practice and may have shaped the causal structures identified. While changes over time were discussed implicitly, a formal trend‐over‐time exercise could have strengthened the grounding of dynamic hypotheses. Accordingly, while the identified feedback loops suggest how system dynamics may evolve, the CLD does not claim to represent specific behavioural trajectories over time, reflecting the study's exploratory and participatory focus. Future studies would benefit from explicitly incorporating reference modes. In addition, defining system boundaries through participant consensus ensured contextual relevance but may have limited the inclusion of external drivers not identified within the workshop discussions. Although already deemed a strength, the extensive network already in place (enabling recruitment) could represent a limitation to others replicating a similar exercise without such networks in place. In a different context/setting, the time required to build relationships might be a barrier [76]. Finally, our intervention list requires further development to detail the implications for responsible agents identified, and to shape optimal policy and practice designs. For example, reviewing good practices internationally, and including where actions align with current political interests. This was beyond the scope of this study.

### 4.5 Policy and Practice Implications and Recommendations

Our study illustrates a range of policy and practice actions to realise the potential of the PFF as a social innovation exemplar to deliver (blue) food system transformation. We have identified changes required from multiple food system actors, from different levels of government, national to local, plus institutions, private sector (business, social enterprise), public sector (schools, education and health/nutrition professionals), producers (fishers) and data and standards bodies. Sustained coordination/leadership of and collaboration between these actors is essential [28, 52]. Furthermore, there needs to be optimised integration of demand and supply chain actions across the system, whereby educational interventions (promoting nutrition and fish literacy) are placed within a strong infrastructural and logistical/supply chain context for optimal impact [53]. For example, nutritional policies seem currently to act as a break/inhibitor but with potential for disruption/change. There is scope, therefore, to educate nutrition/dietetic practitioners better in systems thinking [20] so they can contribute to improved nutritional (policy) outcomes within broader food systems resilience and economic development contexts (with the potential barriers this might present). One of the biggest challenges is economic viability—affordability for schools and fair price for small boat fishers—requiring careful navigation with alignment of pricing structures, procurement policies and funding streams.

## 5 Conclusion

This paper demonstrates how systems thinking and expert elicitation approaches can encourage dynamic dialogue, which can in turn shift narratives and mindsets and potentially disrupt supply chains. Our processes have brought stakeholders together to collaborate towards a shared goal and our PFF innovation serves as a ‘blueprint’, which could be adapted to different contextual or geographical settings. The CLD reveals not only the complexity of interactions, but it also identifies opportunities for targeted, meaningful interventions that have potential to benefit all players across the food system—health practitioners, policy makers, researchers, producers and manufacturers alike.

## Author Contributions

Pettinger Clare designed the research, Pettinger Clare/Parsons Kelly facilitated workshops and analysed/interpreted the data and drafted the manuscript. Irazu Yanaina Chavez‐Ugalde brought CLD/GMB expertise following Workshop 1, and contributed to facilitation, analysis/interpretation and write up. Hunt Louise supported delivery of Workshop 2, synthesis and write up. Wagstaff Carol acquired funding, made substantial contributions to the conception of the research and critically revised the manuscript for important intellectual content. All authors read and approved the final manuscript.

## Ethics Statement

This project was approved (Ref: 5208) by the Faculty of Health Research Ethics & Integrity Committee at the University of Plymouth.

## Conflicts of Interest

The authors declare no conflicts of interest.
